# Genome-Wide Analysis, Characterization, and Expression Profile of the Basic Leucine Zipper Transcription Factor Family in Pineapple

**DOI:** 10.1155/2020/3165958

**Published:** 2020-05-11

**Authors:** Yanhui Liu, Mengnan Chai, Man Zhang, Qing He, Zhenxia Su, S. V. G. N. Priyadarshani, Liping Liu, Guanxi Dong, Yuan Qin

**Affiliations:** State Key Laboratory of Ecological Pest Control for Fujian and Taiwan Crops, Key Laboratory of Genetics, Breeding and Multiple Utilization of Crops, Ministry of Education, Fujian Provincial Key Laboratory of Haixia Applied Plant Systems Biology, Center for Genomics and Biotechnology, College of Life Science, College of Plant Protection, Fujian Agriculture and Forestry University, Fuzhou 350002, Fujian Province, China

## Abstract

This study identified 57 basic leucine zipper (bZIP) genes from the pineapple genome, and the analysis of these bZIP genes was focused on the evolution and divergence after multiple duplication events in relation to the pineapple genome fusion. According to bioinformatics analysis of a phylogenetic tree, the bZIP gene family was divided into 11 subgroups in pineapple, *Arabidopsis*, and rice; gene structure and conserved motif analyses showed that bZIP genes within the same subgroup shared similar intron-exon organizations and motif composition. Further synteny analysis showed 17 segmental duplication events with 27 bZIP genes. The study also analyzed the pineapple gene expression of bZIP genes in different tissues, organs, and developmental stages, as well as in abiotic stress responses. The RNA-sequencing data showed that *AcobZIP57* was upregulated in all tissues, including vegetative and reproductive tissues. *AcobZIP28* and *AcobZIP43* together with the other 25 bZIP genes did not show high expression levels in any tissue. Six bZIP genes were exposed to abiotic stress, and the relative expression levels were detected by quantitative real-time PCR. A significant response was observed for *AcobZIP24* against all kinds of abiotic stresses at 24 and 48 h in pineapple root tissues. Our study provides a perspective for the evolutionary history and general biological involvement of the bZIP gene family of pineapple, which laid the foundation for future functional characterization of the bZIP genes in pineapple.

## 1. Introduction

Transcription factors (TFs) play important roles in the growth and development of plants [1]. When plants are subjected to low temperature, drought, salt stress, or exogenous hormones, TFs are induced to bind to their corresponding *cis*-elements through a series of signal transduction steps to activate or inhibit gene expression [2]. Genes in the ERF family encode transcriptional regulators with a variety of functions involved in the developmental and physiological processes in plants [3]. GmERF5, for example, could bind to the GCC-box element and act as a repressor of gene transcription; the expression of *GmERF5* was significantly induced by *P. sojae*, ethylene (ET), abscisic acid (ABA), and salicylic acid (SA) [4]. WRKY70 and WRKY54 cooperate as negative regulators of stomatal closure and osmotic stress tolerance in *Arabidopsis*, suggesting that they have an important role in plant defense and abiotic stress signaling [5]. OsbZIP62 is involved in the ABA signaling pathways and positively regulates rice drought tolerance by regulating the expression of genes associated with stress [6]. The identification and functional depiction of TFs are essential for the reconstruction of transcriptional regulatory networks [7].

The basic leucine zipper (bZIP) TF family is one of the largest and most diverse TF families [8] and is prevalent in plants, animals, and microorganisms [9]. The bZIP TFs are involved in various biological processes under normal conditions, such as cell elongation [10], organ and tissue differentiation [11], somatic embryogenesis [12], seed-related processes [13], and flower development [14]. People found that plants overexpressing *ZmbZIP22* showed reductions in the size of starch granules and the size and weight of seeds, reduced amylose content, and alterations in the chemical structure of starch granules [15]. The potato transcription factor StbZIP61 functions in concert with StNPR3L to regulate the temporal activation of SA biosynthesis, which contributes to SA-mediated immunity against *P. infestans* infection [16]. ABI5 expression is regulated by ABF3, which could contribute to salt stress tolerance in *Arabidopsis* [17]. Previous studies reported that in *Arabidopsis*, *ATbZIP63* is regulated by ABA, suggesting the involvement of this gene in the cross talk between carbohydrate and ABA-mediated responses [18]. Further studies found that *ATbZIP10* could regulate PR gene expression, cell death, and basal defense response [19, 20]. People also found that *OsbZIP23* plays an important role in ABA signaling and biosynthesis in rice and overexpressed *OsbZIP23* shows improved tolerance to salt and drought stress [21], and overexpression of the *OsbZIP71* enhances tolerance to drought stress [22]. *CsbZIP40* positively regulates SA synthesis, and the expression level of *CsNPR1* was also regulated by *CsbZIP40* expression in sweet orange [23].

The bZIP TFs represent a family of proteins that includes the conserved bZIP domain, which possesses a conserved 40- to 80- amino acid bZIP domain with two structural features: a basic DNA-binding region and a leucine zipper dimerization motif [24]. The basic region of 16 amino acid residues with an invariant N-x7-R/K motif is highly conserved and responsible for nuclear localization and DNA binding [25, 26]. The members of the bZIP TF family have been reported in many eukaryotic genomes, such as the 75 distinct members of the bZIP gene family which were identified in *Arabidopsis* [11], 89 in rice [13], 160 in soybean [27], 88 in poplar [28], 55 in grape [29], 64 in cucumber [26], and 125 maize [30]. Different sizes of the bZIP gene family result in functional variations that can target different kinds of biotic and abiotic stresses [31].

Pineapple (*Ananas comosus* (*Linn.*) *Merr*) is a perennial monocot crop and is widely grown in tropical and subtropical regions. The genome sequencing of pineapple was completed in 2015 [32], which laid the foundation of the functional study of genes and their relationships. The genome-wide association studies of some TF families have been conducted and reported in pineapple, such as SBP [33], WRKY [34], and DOF [35]. However, the bZIP gene family of pineapple has not yet been carried out.

In this study, a total of 57 bZIP genes were found, which were categorized into 11 subgroups on the basis of their phylogenetic relationships and were located to specific chromosomes. This study displayed the intron-exon organizations, motif compositions, gene duplications, and phylogenetic and synteny relationships of the bZIP gene family members in pineapple. The expression patterns of bZIP genes in different tissues and various kinds of stress responses were identified. The results provide valuable information on the evolutionary history and biological functions of the bZIP gene family of pineapple.

## 2. Materials and Methods

### 2.1. Identification of bZIP Gene Family Members in Pineapple

We downloaded pineapple and rice protein sequences from the Phytozome portal (https://phytozome.jgi.doe.gov/pz/portal.html). The *Arabidopsis* proteins were downloaded from the website of TAIR (https://www.arabidopsis.org/). The conserved bZIP domain profile Hidden Markov Models (HMM) (PF00170, PF03131, and PF07716) were downloaded from the Pfam protein family database (http://pfam.xfam.org) [36]. We screened the homologous protein sequence using the BLASTP and HMMER (v.3.0) software with a threshold of *e* value < 1*e*^−5^. Finally, to confirm that the selected candidate gene sequences belong to the bZIP gene family, we used the domain analysis programs of Pfam and SMART (Simple Modular Architecture Research Tool: http://smart.embl-heidelberg.de/) [37]. The isoionic point (IP) and molecular weights of bZIP genes were calculated using the ExPASy server (https://www.expasy.org/vg/index/Protein). To investigate the possible role of *cis*-regulatory elements in the promoter regions of bZIP genes, a 2 kb region upstream of the bZIP gene sequences was used.

### 2.2. Sequence Alignments and Phylogenetic Analysis

All sequences were aligned using the MUSCLE program (http://www.ebi.ac.uk/Tools/msa/muscle/) [38]. All parameters were set as default. An unrooted phylogenetic tree was constructed using the MEGA (Molecular Evolutionary Genetics Analysis, v.6.0) software (the neighbor-joining method) [39] and the RAxML program (the maximum likelihood method). The bootstrap test was performed with 1000 iterations. The tree was displayed using the Interactive Tree of Life (http://itol.embl.de/index.shtml) [40].

### 2.3. Motif and Gene Structure Analyses

The MEME (Multiple EM for Motif Elicitation, v.4.9.0) software was employed to identify the bZIP gene family conserved motifs [41]. The width of the motifs was between 10 and 100 aa, and the number of motifs was 20. For further understanding of the gene structure of the bZIP gene family of pineapple, we downloaded the pineapple GFF (Generic Feature Format) file from Phytozome (v.12.1) and wrote a Perl script to arrange the GFF annotation results in the order of the bZIP genes in pineapple in the phylogenetic tree. Gene structure analysis of the bZIP gene family was performed using the Gene Structure Display Server (http://gsds.cbi.pku.edu.cn/).

### 2.4. Synteny Analysis

We used the BLASTP program to search homologous gene pairs among pineapple, rice, and *Arabidopsis* (*e* value < 1*e*^−5^, max_target_seqs = 5). These gene pairs were used as the input for bio-pipeline-master to calculate Ka, Ks, and Ka/Ks values [42]. The chromosome location and gene collinearity in pineapple were analyzed using the Circos software [43]. The syntenic maps between pineapple and other selected species were constructed using JCVI (https://github.com/tanghaibao/jcvi).

### 2.5. Expression Profiles Based on the Estimation of Expression Levels from RNA-seq Data

To investigate the expression pattern of the bZIP genes in pineapple development, we obtained the estimated expression levels and RPKM (reads per kilobase per million reads) values for each AcobZIP from nine different tissues and different developmental stages of each tissue. Four stages of the sepal, three stages of the petal, five stages of the stamen, and seven stages of the ovule samples were downloaded from the Discovery Environment (https://de.iplantcollaborative.org/de/?type=data&folder=/iplant/home/cmwai/coge_data/Pineapple_tissue_RNAseq). In total, the ovules of seven different developmental stages were collected, including stage 1, bud width < 5 mm; stage 2, bud width 5–8 mm; stage 3, bud width 8 mm; stage 4, bud width > 8 mm to petals invisible; stage 5, just showing petals; stage 6, with 1 mm petals visible; and stage 7, >2 mm petals visible to preflowering. The five stages of stamen development are as follows: stage 1, bud width < 5 mm; stage 2, bud width 5–8 mm; stage 3, bud width > 8 mm to petals invisible; stage 4, just showing petals; and stage 5, buds just showing petals to the buds with 1 mm petal visible. RNA-sequencing (RNA-seq) data for tissues such as the flower, leaf, root, and six stages of the fruit were downloaded from the Pineapple Genomics Database (http://pineapple.angiosperms.org/pineapple/html/download.html). The heatmap of bZIP gene expression profiles was generated by pheatmap (a package of R software).

### 2.6. Plant Material and Treatments

We collected one-month-old pineapple plants grown in a liquid medium. The seedlings were obtained by tissue culture from Qin Lab, Fujian Agriculture and Forestry University, Fujian, China (http://www.qinlab.net) [44]. Abiotic stress treatments included low-temperature (cold), high-temperature (heat), drought, and high-salt conditions. For salt and drought stress treatment, one-month-old pineapple plants were treated with 150 mM NaCl and 400 mM mannitol, respectively, for 0, 6, 12, 24, and 48 h. Heat treatment was carried out in a growth chamber at 45°C, with 70% humidity, and light availability of 60–70 mmol photons m^−2^ s^−1^ under a 16 h light/8 h dark photoperiod. Cold treatment was carried out in a growth chamber at 4°C under the conditions mentioned above. The leaves were harvested at the indicated time points for the preparation of total RNA. Samples were collected from both the stressed and normal plants (those not exposed to stress treatment), which were used as controls. The collected samples were immediately stored in liquid nitrogen before total RNA extraction.

### 2.7. RNA Extraction and qRT-PCR

According to the manufacturer's protocol, we extracted the total RNA using an RNA plant extraction kit (Omega Bio-tek, Shanghai, China). According to the supplier's instructions to use AMV reverse transcriptase (Takara), 1 *μ*g purified total RNA was reverse transcribed to cDNA in a 20 *μ*l reaction volume [45]. To detect the transcript levels of the selected bZIP genes, RT-PCR was performed with gene-specific primers according to the manufacturer's instructions on the Bio-Rad RT-PCR system (Foster City, CA, USA). The primers used in this study are provided in Table S7. The internal reference gene we used is AcoPP2A (XM020244640). Relative expression was then considered the *Δ*Cp (according to Roche Diagnostics software LC480) between each gene and the average of controls. Experiments were done in triplicate. Average *Δ*Cp was calculated from the three replicates and compared between treatment and control. For this, the *ΔΔ*Cp and the *P* value relative to the standard *t*-test were calculated. The RT-PCR program was set at 95°C for 30 s, 40 cycles of 95°C for 5 s, 60°C for 34 s, and 95°C for 15 s. Three technical replicates and at least three independent biological replicates were used in each treatment [46].

## 3. Results

### 3.1. Identification of bZIP Genes in Plants

In recent years, the increasing number of whole-genome sequencing projects provided researchers important basis to study the development of the key system and evolution of gene families in plants. A total of 57 bZIP gene family members were identified in pineapple and named from *AcobZIP1* to *AcobZIP57* (Table S1). The encoded proteins of AcobZIP genes varied substantially in size, sequences, and physicochemical properties. The IP and molecular weights of the bZIP genes are presented in Table S1. The lowest IP value was 4.66 for *AcobZIP38*, whereas the highest IP value was 10.26 for *AcobZIP49*. According to the results of relative molecular mass distribution, bZIP proteins mass varies from 13.731 (AcobZIP52) to 71.643 (AcobZIP34) kDa.

### 3.2. Phylogenetic Classification of the bZIP Gene Family of Pineapple

**Figure 1:**
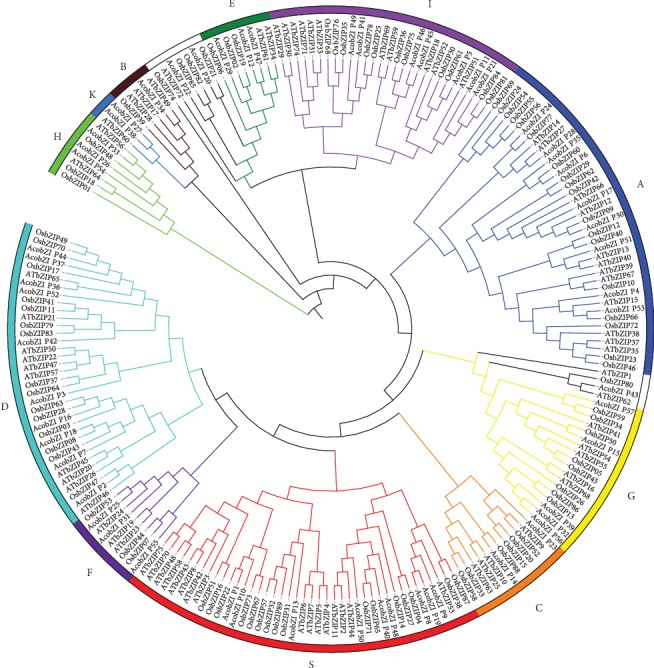
Maximum likelihood phylogenetic tree of the bZIP gene family of pineapple, *Arabidopsis*, and rice.

To analyze the phylogenetic relationship of bZIP TFs between pineapple, *Arabidopsis*, and rice, multiple sequence alignments were carried out according to full-length amino acid sequences using the MUSCLE software. An unrooted phylogenetic tree of pineapple, *Arabidopsis*, and rice was constructed using the RAxML program. On the basis of the phylogenetic tree (Figure 1), the bZIP gene family can be divided into 11 subgroups. Ten subgroups (A, B, C, D, E, F, G, H, I, and S) are similar to *Arabidopsis* and name the groups following *Arabidopsis* [11], and the K subgroup is the same as that in soybean [27], suggesting that the bZIP gene family was conserved in evolutionary history. The interspecies clustering indicates a parallel evolution of bZIP TFs in three species, and the homologous bZIP proteins in the same subgroup develop similar functions. However, *ATbZIP62*/*AcobZIP43*/*OsbZIP80* and *ATbZIP72*/*AcobZIP20*/*AcobZIP22*/*OsbZIP21*/*OsbZIP74*/*OsbZIP82*/*OsbZIP85*/clades form two small unique clades in the phylogenetic tree and may have had independent evolutionary trajectories from other clades.

### 3.3. Conserved Motifs and Gene Structure Analysis of the bZIP Gene Family of Pineapple

**Figure 2:**
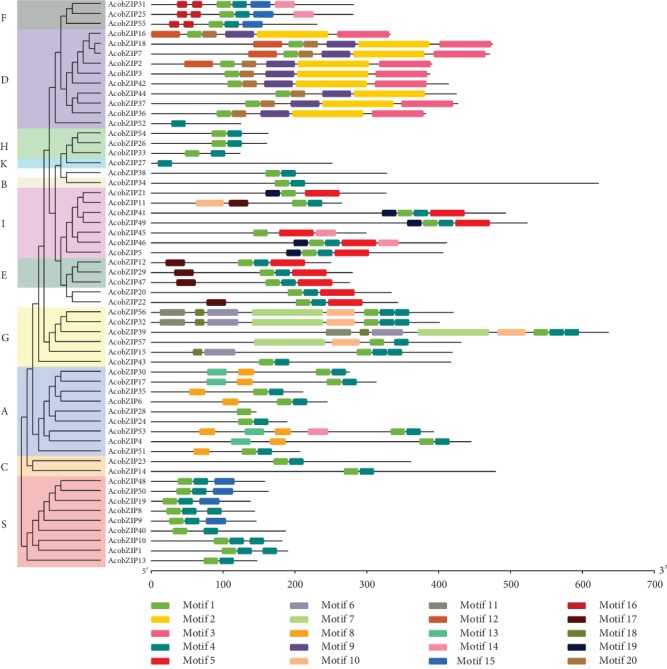
Motif composition of pineapple bZIP proteins. The motifs, numbers from 1 to 20, are displayed in different colored boxes.

A total of 20 conserved motifs have been identified in AcobZIP protein sequences. As exhibited in Figure 2, most of the AcobZIP proteins within the same subgroup displayed similar motif compositions, but there was high variance among the different subgroups. All the subgroups contained motif 1, which was annotated as the bZIP domain. Motifs 2, 3, and 9 were unique to subgroup D, whereas motif 5 was specific to subgroups E and I. Subgroup G had two specific motifs, including motifs 6 and 7. We found that motifs 8 and 13 were unique in subfamily A. The clustered AcobZIP pairs, *AcobZIP26*/*33*/*54* in subgroup H, showed a highly similar motif distribution, indicating that they might have similar functions. The similar motif arrangements among AcobZIP proteins within the same subgroups indicated that the protein architecture is conserved within a specific subgroup.

**Figure 3:**
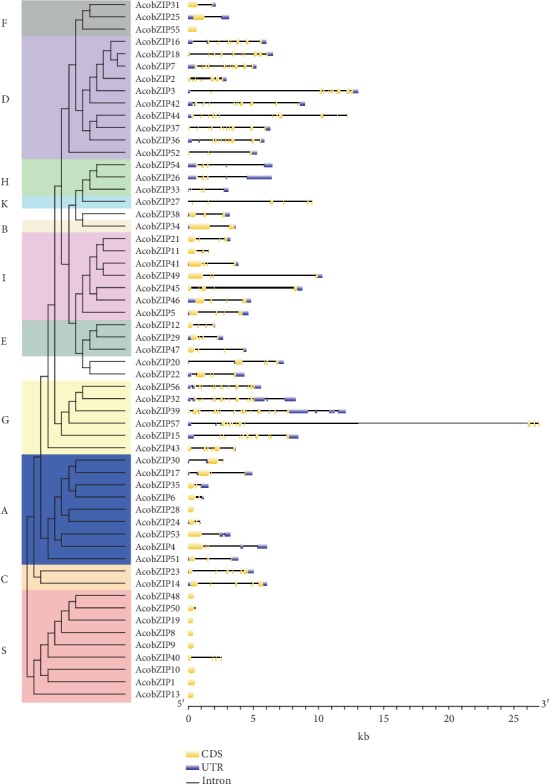
Exon-intron structure of pineapple bZIP genes.

As a kind of evolutionary relic, the intron-exon arrangement carries the imprint of the evolution of the bZIP gene family [47]. The Gene Structure Display Server was employed to analyze the structural organization of the intron-exon arrangement of the bZIP gene family of pineapple. Genes within the same group usually have a similar gene structure. As shown in Figure 3, all AcobZIP genes possessed 1-15 exons. For example, most of the subgroup I members contained 4 exons and 3 introns except *AcobZIP11* which contains three exons and two introns and *AcobZIP45* which contains six exons and five introns; most of the subgroup D members had 10 exons and 9 introns; subgroup C had 6 exons and 5 introns; subgroup E had 4 exons and 3 introns; subfamily F clade had 1 or 2 exon and 1 intron; and most of the subgroup S clade had only 1 exon without the intron. Overall, the conserved motif compositions and similar gene structures of the bZIP members in the same group, together with the phylogenetic analysis results, could strongly support the reliability of the group classifications.

### 3.4. Synteny Analysis of the bZIP Gene Family of Pineapple

On the basis of the pineapple genome sequence, the bZIP gene location mapping revealed that AcobZIP gene family members were unevenly distributed on the 23 chromosomes in pineapple, except for chromosomes LG15 and LG16. On the contrary, *AcobZIP54* and *AcobZIP56* were located on scaffolds 1613 and 2165, respectively, which have not been mapped onto any chromosome. We found that three genes were mapped onto LG1, which was the longest chromosome; LG2 and LG18 contained the largest number of bZIP genes (four) in pineapple, whereas the length of LG2 and LG18 was shorter than that of LG1 (Table S1). Therefore, the results revealed that there is no positive correlation between the length of chromosomes and the number of bZIP genes on a chromosome.

**Figure 4:**
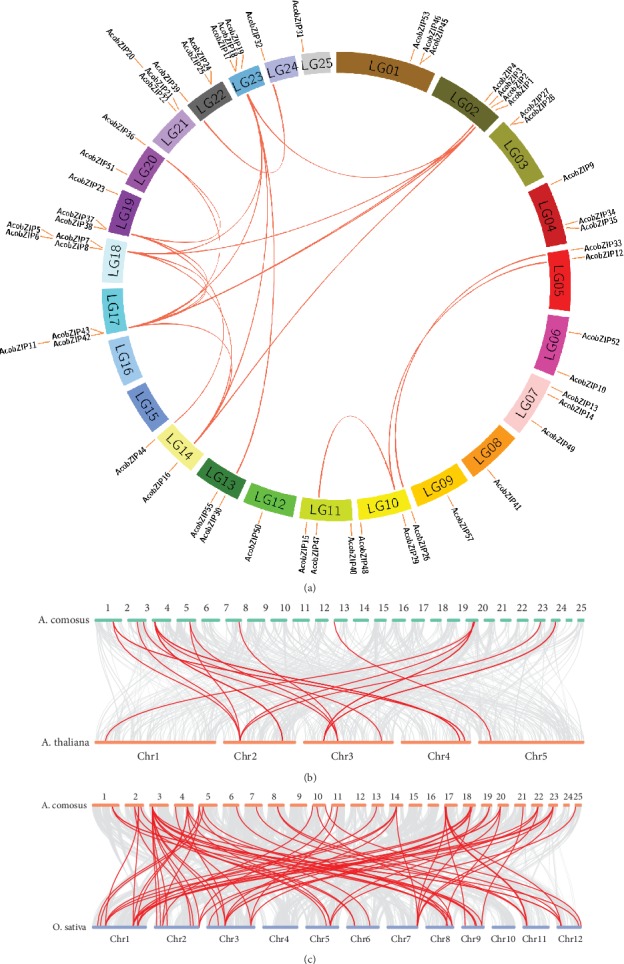
Gene location and synteny in pineapple. (a) All bZIP genes were mapped to their respective chromosomes in pineapple in a circular diagram using Circos. (b) Synteny analysis of bZIP genes between pineapple and *Arabidopsis*. (c) Synteny analysis of bZIP genes between pineapple and rice.

Seven tandem duplication events were observed in the pineapple genome, including *AcobZIP6*/*AcobZIP7*, *AcobZIP17*/*AcobZIP18*, *AcobZIP24*/*AcobZIP25*, *AcobZIP27*/*AcobZIP28*, *AcobZIP37*/*AcobZIP38*, *AcobZIP42*/*AcobZIP43*, and *AcobZIP45*/*AcobZIP46*. In addition, 24 pairs of segmental duplication events with 20 bZIP genes in pineapple were identified (Figure 4(a) and Table S2). These results indicated that some AcobZIP genes were possibly generated by gene duplication and segmental duplication events, which acted as a major driving force for the evolution of AcobZIP.

We constructed two comparative syntenic maps of pineapple associated with *Arabidopsis* and rice to further infer the phylogenetic mechanism of the bZIP TF family (Figures 4(b) and 4(c)). A total of 16 AcobZIP genes showed a syntenic relationship with those in *Arabidopsis* and 73 in rice (Tables S3 and S4). The number of collinear gene pairs between pineapple and rice is greater than that between pineapple and *Arabidopsis*. Therefore, we hypothesize that both pineapple and rice (monocotyledon) share a higher number of collinear gene pairs compared to monocots and dicots. Ka/Ks values can be used to predict selection pressure for replicating genes. The Ka, Ks, and Ka/Ks values of the bZIP gene pairs were calculated for *Arabidopsis*, rice, and pineapple. Most of the segmental duplicated AcobZIP gene pairs showed a Ka/Ks ratio < 0.3, with the highest in the *AcobZIP32*-*AcobZIP56* pair (Ka/Ks value = 0.92). All the Ka/Ks ratios < 1 suggest that they likely underwent a strong purifying positive selection during evolution. The Ka and Ks values can be used to predict the selective pressure on duplicated genes, such that a Ka/Ks ratio > 1 indicates positive selection, Ka/Ks ratio = 1 indicates neutral selection, and Ka/Ks ratio < 1 shows purifying selection [48].

### 3.5. Expression Pattern of bZIP Genes in Different Tissues of Pineapple

**Figure 5:**
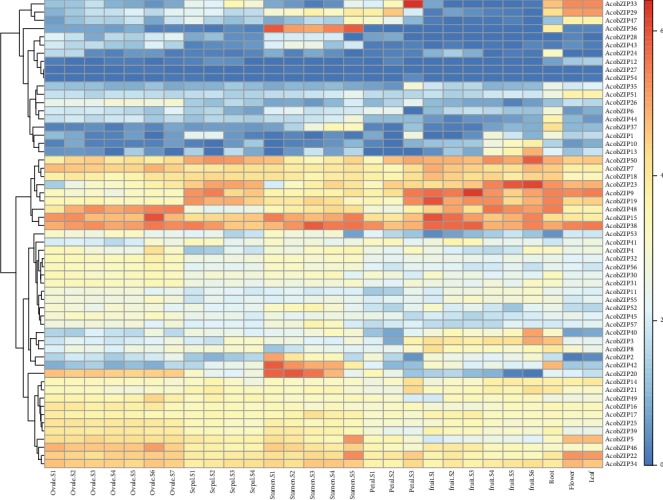
Expression profiles of bZIP genes in different pineapple tissues. The color bar shows the gene expression with log2(FPKM + 1).

**Figure 6:**
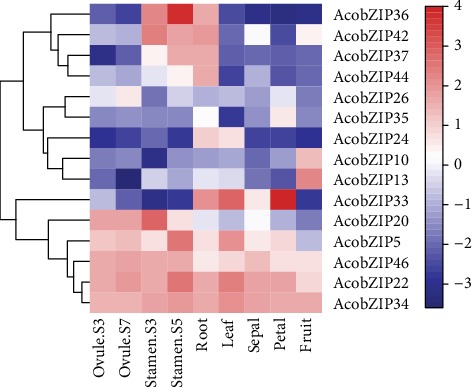
The qPCR validation results of bZIP genes in pineapple tissues.

To further analyze the expression pattern of pineapple, we explored the developmental gradient and different tissues of pineapple. Most of the AcobZIP genes were expressed in all the tissues and had no significant changes (Figure 5 and Table S5), whereas the expression levels of some genes varied differently among different tissues. For example, *AcobZIP29* showed high expression levels in the root, flower, leaf, petal, and sepal but relatively low expression levels in other tissues. *AcobZIP37* was abundantly expressed in the roots and stamen but expressed at lower levels in other tissues. *AcobZIP26* expression gradually increased from ovule S1 to S7 in pineapple, indicating that it might be involved in ovule development. *AcobZIP36* and *AcobZIP20* showed high expression levels in stamen tissue, indicating that they might be associated with stamen development. *AcobZIP38* displayed high expression levels in all tissues in pineapple. In addition, 19 genes showed lower expression levels in all organs than any other gene. Ten bZIP genes showed high expression levels in all tissues. Further, 11 genes showed high expression levels in the ovule; 6 genes showed high expression levels in the flower; and 7 genes showed high expression levels in the stamen. Tissue-specific RNA-seq results demonstrated that the members of the bZIP gene family might be involved in the growth and development of different tissues or organs in pineapple. The expression levels of tissue-specific bZIP genes were examined using qPCR to verify the accuracy of these data. As shown in Figure 6, *AcobZIP5*, *AcobZIP22*, *AcobZIP34*, and *AcobZIP46* displayed high expression levels in all tissues; *AcobZIP36*/*AcobZIP37*/*AcobZIP42* showed the highest expression levels in the stamen and root. The transcript levels of *AcobZIP26* and *AcobZIP35* were low in all tissues. The results are consistent with the gene transcript abundance data presented in Table S5.

### 3.6. Expression Profiles of bZIP Genes against Abiotic Stress

To explore the mechanisms of bZIP genes in response to abiotic stress, we searched for 15 stress-related *cis*-elements in promoter regions, which is 2 kb upstream in pineapple genes, such as ABRE, ERE, GARE, and W-box. The results showed that more than three different *cis*-elements were located in the promoters of most *bZIP* genes. Some elements were detected in more than one copy in the promoter region. For example, the promoter of *AcobZIP9* contained five copies of ABRE sequences and the promoter of *AcobZIP35* contained three copies of W-box sequences (Table S6). ABRE is a major ABA-dependent gene expression *cis*-regulatory element that is ubiquitous in the bZIP genes in pineapple, and W-box is an important class of *cis*-acting elements, mainly in the promoter of plant defense response-related genes [49, 50]. The results indicated that bZIP gene family members might play an important role in response to some kinds of stress.

**Figure 7:**
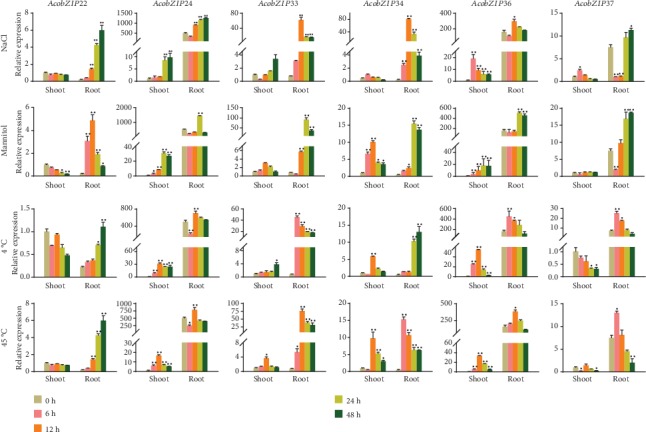
Expression levels of six bZIP genes in response to different stress treatments in pineapple.

To further confirm if AcobZIP gene expression was influenced by different kinds of abiotic stress, six bZIP genes (*AcobZIP22*, *AcobZIP24*, *AcobZIP33*, *AcobZIP34*, *AcobZIP36*, and *AcobZIP37*) were selected to examine the expression profiles under cold, heat, salt, and drought treatments for 0, 6, 12, 24, and 48 h in “MD2” variety of pineapple. Two tissues (the root and shoot) were used to examine for RNA extraction and then perform qRT-PCR. As shown in Figure 7, the expressions of the six genes were induced by four kinds of abiotic stress in the root at least at one time point. The transcript levels of some genes were not changed obviously in the shoot. For example, *AcobZIP37* expression was not induced under drought stress in the shoot. In addition, the expression of some genes could be induced by abiotic stress in the root and shoot, but their expression levels were much lower in the shoot than in the root, such as *AcobZIP33* and *AcobZIP34* in response to all kinds of abiotic stress. On the contrary, some genes showed high expression levels in both the shoot and the root in response to abiotic stress, such as *AcobZIP22*, whose expression was significantly induced under cold stress, and the transcript level in the shoot and root was comparable.

## 4. Discussion

TFs act as the key controller of major developmental processes and have indispensable roles in a variety of biological processes [51–53]. The transcriptional regulation of gene expression affects many important cellular processes, such as morphogenesis, environmental stress responses, and signal transduction [54]. The bZIP TFs are found in all eukaryotes, and they form one of the largest families of dimerizing TFs. In plants, the bZIP motif regulates various processes, including resistance against pathogens, light signaling, abiotic stress responses, seed maturation, and flower development. The bZIP gene family is widely studied in different plant species but not in pineapple.

### 4.1. bZIP Genes Are Highly Conserved in Pineapple

The number of bZIP gene family members varies in different species, and it is especially less in lower plants. A total of 17 bZIP genes containing typical bZIP domain were identified in *Chlamydomonas reinhardtii* [55], and 37 bZIP genes were identified in *Physcomitrella patens*, which was the earliest land plant moss [13]. However, in angiosperms, the number of bZIP genes in most plant genomes was more than 50 [11, 27, 30, 56, 57]. There were 57 bZIP TF-encoding genes present in the pineapple genome. The higher number of bZIP genes in a particular genome mainly resulted from the polyploidization or whole-genome duplication (WGD) event [58]. Gene duplication (GD) provides the evolutionary potential for generating novel genes and functions [59]. More intense gene expansion of TF families undergoing angiosperm possibly reflects the ability of flowering plants to efficiently adapt to different unstable environmental conditions [13].

The bZIP gene family of pineapple was compared with the model plant *Arabidopsis*. In many plants, the members of the bZIP gene family are classified into subgroups according to sequence similarity, conserved motifs, and gene structure [60]. The phylogenetic tree suggested an evolutionary insight into bZIP genes among species. In this study, 57 bZIP genes in pineapple were divided into 11 subgroups. For further investigation, the bZIP genes were analyzed for their protein sequences to find the common motif of amino acids. As a motif refers to a pattern in a protein structure formed by the spatial arrangement of amino acids, there would be some proteins showing structural and functional similarity with others. Additionally, motif composition shows the reliability of the phylogeny. As a big family, the bZIP genes were divided into 20 motifs in pineapple. All the subgroups contained motif 1, which was annotated as the bZIP domain. Conserved motif results indicated that all the bZIP TFs shared the conservative bZIP domain, and each subgroup harbored similar motifs. A total of 75 bZIP genes in *Arabidopsis* can be clustered into 10 bZIP subgroups, among which subgroup D is involved in defense against pathogens [61]. In our study, we found that 10 genes (*AcobZIP2*, *AcobZIP3*, *AcobZIP7*, *AcobZIP16*, *AcobZIP18*, *AcobZIP36*, *AcobZIP37*, *AcobZIP42*, *AcobZIP44*, and *AcobZIP52*) belong to subgroup D with motifs 2 and 9, indicating that these genes may also participate in defense against pathogens.

It is considered that phylogenetic analyses must be accompanied by the intron-exon arrangement in a gene family. Gene structure analysis revealed different clusters of genes divided on the basis of structural similarity among the bZIP genes in pineapple. Few genes lacked introns, whereas some did not have UTRs, showing gene structural diversity for functional studies for important bZIP candidate genes. People [62] also examined the intron-exon arrangement of the 112 *MdbZIP* genes, because intron-exon diversification of gene family members is known to have played a vital role in the evolution of several gene families. Scientists [25] already showed that ∼20% of the genes had no introns. The same results were also reported in rice and sorghum, with almost the same percentage of genes having no intron. Gene structure and conserved motif analyses showed that the same subfamily harbored similar intron-exon organizations and conserved motifs, indicating that the relationship of bZIP in the same group was closer during the gene evolution process [63].

Synteny analysis revealed that AcobZIP genes were possibly generated by gene duplication and segmental duplication events, which had a major impulsive effect on the evolution of AcobZIP. As the Ka/Ks ratio is used to estimate the balance between neutral mutations, purifying selection and beneficial mutations act on a set of homologous protein-coding genes. Therefore, we calculated Ka/Ks for bZIP genes. The Ka, Ks, and Ka/Ks values of the bZIP gene pairs of *Arabidopsi*s, rice, and pineapple were calculated and compared. The bZIP genes also undergo a strong purifying positive selection in *Arabidopsis*, rice, and soybean [27]. Interestingly, the AcobZIP gene pairs also show tandem and segmental duplication events under similar evolutionary pressure, with all the Ka/Ks ratios < 1.

### 4.2. Functional Study of bZIP Genes

The bZIP TFs have been suggested to be widely participating in the integration and physical development of many organs and tissues, such as germination, seed maturation [64], and flower initiation and development [65, 66]. To determine the bZIP gene expression in different tissues, RNA-seq data were analyzed to witness bZIP gene tissue-specific expression in pineapple. A study on the bZIP gene family of grapes concluded that VvbZIP showed a peculiar expression in flower organs; in particular, *VvbZIP54* was highly expressed in Flower-FB, Flower-F, stamen, and pollen, which is associated with our recent results of bZIP in pineapple. Collectively, 10 genes showed high expression levels in all tissues. On the contrary, *AcobZIP28* and *AcobZIP43* together with other 25 bZIP genes were not expressed in any tissues, showing their specificity for marker selection with other highly expressed genes. *AcobZIP14* and *AcobZIP21* were moderately expressed in all tissues along with nine other bZIP genes. According to the previous study in the bZIP genes in apple, most of the genes showed expression in all the tissues studied (stems, roots, young leaves, mature leaves, flower buds, young fruits, seeds of young fruits, and mature fruits) with variable levels, and they all showed a definitive expression in the seeds of fruits, except for *MdbZIP91* [25]. Previous results support our study of bZIP expression, resulting in the expression of almost 15 genes in many tissues.

Plants and crops are frequently affected by environmental stimuli such as abiotic stress, drought stress, and high temperature. Some evidence has suggested that bZIP proteins are widely involved in signaling and responses to abiotic/biotic stimuli [56, 67]. There is appreciable evidence that bZIP genes are the key regulators of many central developmental and physiological processes in several plant species [19]. Here, we performed an experiment to know more about the bZIP genes in pineapple and their response against abiotic stress. We selected six genes to test against salt (NaCl), drought (mannitol), cold, and heat stress for 0, 6, 12, 24, and 48 h in root and shoot tissues. A considerably significant response was observed for *AcobZIP24* against all kinds of abiotic stress at 24 and 48 h in pineapple root tissues, followed by *AcobZIP36*, which was also upregulated against abiotic stress at high-level treatments. *AcobZIP22* and *AcobZIP37* did not respond to abiotic stress, as they should have. On the basis of our studies, we can conclude that *AcobZIP24* and *AcobZIP36* are actually representing bZIP genes as they are known as the key regulators of different aspects of developmental studies. Earlier AREB1 was reported to intensify tolerance to drought stress in *Arabidopsis* [49]. It was observed that *OsbZIP16* positively regulates drought resistance in rice [68]. Previous studies showed the expression of six bZIP genes against abiotic stress (drought and salinity) [25], and their findings indicated that all the selected genes did not show a response to abiotic stress, which is contrary to our results. According to Liu et al. [29], some bZIP genes showed significant expression against abiotic stress, which confirms our bZIP gene expression in pineapple. However, they interestingly observed that some upregulated genes elicited by drought stress were downregulated by heat stress. Our results of bZIP genes against abiotic stress confirm the previous observations about bZIP in different plant species. The TFs regulate transcription by binding to *cis*-acting elements in the promoter region, conferring resistance against pathogens in plants [69]. In our study, most of the AcobZIP genes contained *cis*-elements related to drought resistance, such as ABRE and W-box. It can be inferred that these genes are also involved in drought stress. This study provides the basis for an in-depth study of the bZIP gene family of pineapple in terms of stress and plant development.

## 5. Conclusion

An objective-oriented study was performed to investigate the genome-wide association of the bZIP gene family of pineapple. We identified 57 bZIP gene sequences and performed an analysis of genome data sets, considering phylogeny (*Arabidopsis, rice* and pineapple), gene structures, and conserved motifs, followed by gene location on chromosomes by synteny analysis. The phylogenetic tree of the bZIP gene family was divided into 11 subgroups, and the bZIP proteins showed 20 common motifs. Most of the bZIP genes consisted of two introns and three exons. Using the synteny analysis, 17 segmental duplication events with 27 bZIP genes were identified. On the basis of tissue-specific RNA-seq expression, *AcobZIP38* showed high expression levels for all study tissues, including vegetative and reproductive tissues. Collectively, 10 genes showed high expression levels in all the tissues tested, whereas *AcobZIP28* and *AcobZIP43* together with other 25 bZIP genes showed low expression levels in all tissues. Furthermore, six bZIP genes were subjected to abiotic stress (salt, drought, cold, and heat). A considerably significant response was observed in *AcobZIP24* against all kinds of abiotic stresses at 24 and 48 h in pineapple root tissues, followed by *AcobZIP36*, which was also upregulated against abiotic stress at high-level treatments. These results may provide basic research for a further study of pineapple gene function in response to stress conditions.

## Data Availability

All data generated or analyzed during this study are included in this published article and its supplementary information files.

## Supplementary Materials

Supplementary 1 Fig. S1: 20 motifs of bZIP proteins in pineapple.

Supplementary 2 Table S1: the accession numbers of bZIPs in pineapple, *Arabidopsis*, and rice.

Supplementary 3 Table S2: synteny blocks of bZIP genes within pineapple.

Supplementary 4 Table S3: synteny blocks of bZIP genes between pineapple and *Arabidopsis*.

Supplementary 5 Table S4: synteny blocks of bZIP genes between pineapple and rice.

Supplementary 6 Table S5: the expression profiles of the pineapple bZIP genes in different tissues.

Supplementary 7 Table S6: *cis*-elements in AcobZIP promoter regions.

Supplementary 8 Table S7: primers used in qRT-PCR analysis.

## References

[1] Beckett D. (2001). Regulated assembly of transcription factors and control of transcription initiation. *Journal of Molecular Biology*.

[2] Wray G. A., Hahn M. W., Abouheif E. (2003). The evolution of transcriptional regulation in eukaryotes. *Molecular Biology and Evolution*.

[3] Nakano T., Suzuki K., Fujimura T., Shinshi H. (2006). Genome-wide analysis of the ERF gene family in Arabidopsis and rice. *Plant Physiology*.

[4] Dong L., Cheng Y., Wu J. (2015). Overexpression of GmERF5, a new member of the soybean EAR motif-containing ERF transcription factor, enhances resistance to Phytophthora sojae in soybean. *Journal of Experimental Botany*.

[5] Li J., Besseau S., Toronen P. (2013). Defense-related transcription factors WRKY70 and WRKY54 modulate osmotic stress tolerance by regulating stomatal aperture in Arabidopsis. *The New Phytologist*.

[6] Yang S., Xu K., Chen S. (2019). A stress-responsive bZIP transcription factor OsbZIP62 improves drought and oxidative tolerance in rice. *BMC Plant Biology*.

[7] Zhang H., Jin J., Tang L. (2011). PlantTFDB 2.0: update and improvement of the comprehensive plant transcription factor database. *Nucleic Acids Research*.

[8] Perez-Rodriguez P., Riano-Pachon D. M., Correa L. G., Rensing S. A., Kersten B., Mueller-Roeber B. (2010). PlnTFDB: updated content and new features of the plant transcription factor database. *Nucleic Acids Research*.

[9] Agarwal P., Baranwal V. K., Khurana P. (2019). Genome-wide analysis of bZIP transcription factors in wheat and functional characterization of a TabZIP under abiotic stress. *Scientific Reports*.

[10] Fukazawa J., Sakai T., Ishida S., Yamaguchi I., Kamiya Y., Takahashi Y. (2000). Repression of shoot growth, a bZIP transcriptional activator, regulates cell elongation by controlling the level of gibberellins. *Plant Cell*.

[11] Jakoby M., Weisshaar B., Dröge-Laser W. (2002). bZIP transcription factors in Arabidopsis. *Trends in Plant Science*.

[12] Guan Y., Ren H., Xie H., Ma Z., Chen F. (2009). Identification and characterization of bZIP-type transcription factors involved in carrot (*Daucus carota* L.) somatic embryogenesis. *The Plant Journal*.

[13] Correa L. G., Riano-Pachon D. M., Schrago C. G., dos Santos R. V., Mueller-Roeber B., Vincentz M. (2008). The role of bZIP transcription factors in green plant evolution: adaptive features emerging from four founder genes. *PLoS One*.

[14] Zhang C., Liu J., Zhao T. (2016). A drought-inducible transcription factor delays reproductive timing in rice. *Plant Physiology*.

[15] Dong Q., Xu Q., Kong J. (2019). Overexpression of ZmbZIP22 gene alters endosperm starch content and composition in maize and rice. *Plant Science*.

[16] Zhou X. T., Jia L. J., Wang H. Y. (2018). The potato transcription factor StbZIP61 regulates dynamic biosynthesis of salicylic acid in defense against Phytophthora infestans infection. *The Plant Journal*.

[17] Chang H. C., Tsai M. C., Wu S. S., Chang I. F. (2019). Regulation of ABI5 expression by ABF3 during salt stress responses in Arabidopsis thaliana. *Botanical Studies*.

[18] Matiolli C. C., Tomaz J. P., Duarte G. T. (2011). The Arabidopsis bZIP gene AtbZIP63 is a sensitive integrator of transient abscisic acid and glucose signals. *Plant Physiology*.

[19] Alves M. S., Dadalto S. P., Goncalves A. B., De Souza G. B., Barros V. A., Fietto L. G. (2013). Plant bZIP transcription factors responsive to pathogens: a review. *International Journal of Molecular Sciences*.

[20] Sato F., Kitajima S., Koyama T., Yamada Y. (1996). Ethylene-induced gene expression of osmotin-like protein, a neutral isoform of tobacco PR-5, is mediated by the AGCCGCC cis-sequence. *Plant & Cell Physiology*.

[21] Zong W., Tang N., Yang J. (2016). Feedback regulation of ABA signaling and biosynthesis by a bZIP transcription factor targets drought-resistance-related genes. *Plant Physiology*.

[22] Liu C., Mao B., Ou S. (2014). OsbZIP71, a bZIP transcription factor, confers salinity and drought tolerance in rice. *Plant Molecular Biology*.

[23] Li Q., Jia R., Dou W. (2019). CsBZIP40, a BZIP transcription factor in sweet orange, plays a positive regulatory role in citrus bacterial canker response and tolerance. *PLoS One*.

[24] Hurst H. C. (1995). Transcription factors 1: bZIP proteins. *Protein Profile*.

[25] Zhao J., Guo R., Guo C., Hou H., Wang X., Gao H. (2016). Evolutionary and expression analyses of the apple basic leucine zipper transcription factor family. *Frontiers in Plant Science*.

[26] Baloglu M. C., Eldem V., Hajyzadeh M., Unver T. (2014). Genome-wide analysis of the bZIP transcription factors in cucumber. *PLoS One*.

[27] Zhang M., Liu Y., Shi H. (2018). Evolutionary and expression analyses of soybean basic leucine zipper transcription factor family. *BMC Genomics*.

[28] Evens N. P., Buchner P., Williams L. E., Hawkesford M. J. (2017). The role of ZIP transporters and group F bZIP transcription factors in the Zn-deficiency response of wheat (*Triticum aestivum*). *The Plant Journal*.

[29] Liu J., Chen N., Chen F. (2014). Genome-wide analysis and expression profile of the bZIP transcription factor gene family in grapevine (*Vitis vinifera*). *BMC Genomics*.

[30] Wei K., Chen J., Wang Y. (2012). Genome-wide analysis of bZIP-encoding genes in maize. *DNA Research*.

[31] Li Q., Yu H., Cao P. B. (2015). Explosive tandem and segmental duplications of multigenic families in *Eucalyptus grandis*. *Genome Biology and Evolution*.

[32] Ming R., VanBuren R., Wai C. M. (2015). The pineapple genome and the evolution of CAM photosynthesis. *Nature Genetics*.

[33] Ali H., Liu Y., Azam S. M. (2017). Genomic survey, characterization, and expression profile analysis of the SBP genes in pineapple (*Ananas comosus* L.). *International Journal of Genomics*.

[34] Xie T., Chen C., Li C., Liu J., Liu C., He Y. (2018). Genome-wide investigation of WRKY gene family in pineapple: evolution and expression profiles during development and stress. *BMC Genomics*.

[35] Zou Z., Zhu J., Zhang X. (2019). Genome-wide identification and characterization of the Dof gene family in cassava (Manihot esculenta). *Gene*.

[36] El-Gebali S., Mistry J., Bateman A. (2019). The Pfam protein families database in 2019. *Nucleic Acids Research*.

[37] Letunic I., Doerks T., Bork P. (2012). SMART 7: recent updates to the protein domain annotation resource. *Nucleic Acids Research*.

[38] Edgar R. C. (2004). MUSCLE: multiple sequence alignment with high accuracy and high throughput. *Nucleic Acids Research*.

[39] Tamura K., Stecher G., Peterson D., Filipski A., Kumar S. (2013). MEGA6: Molecular Evolutionary Genetics Analysis version 6.0. *Molecular Biology and Evolution*.

[40] Letunic I., Bork P. (2011). Interactive Tree Of Life v2: online annotation and display of phylogenetic trees made easy. *Nucleic Acids Research*.

[41] Bailey T. L., Boden M., Buske F. A. (2009). MEME SUITE: tools for motif discovery and searching. *Nucleic Acids Research*.

[42] Tang H., Bowers J. E., Wang X., Ming R., Alam M., Paterson A. H. (2008). Synteny and collinearity in plant genomes. *Science*.

[43] Krzywinski M., Schein J., Birol I. (2009). Circos: an information aesthetic for comparative genomics. *Genome Research*.

[44] Priyadarshani S. V. G. N., Hu B., Li W. (2018). Simple protoplast isolation system for gene expression and protein interaction studies in pineapple (*Ananas comosus* L.). *Plant Methods*.

[45] Cai H., Zhao L., Wang L. (2017). ERECTA signaling controls Arabidopsis inflorescence architecture through chromatin-mediated activation of PRE1 expression. *The New Phytologist*.

[46] Cai H., Zhang M., Chai M. (2019). Epigenetic regulation of anthocyanin biosynthesis by an antagonistic interaction between H2A.Z and H3K4me3. *The New Phytologist*.

[47] Pan F., Wu M., Hu W., Liu R., Yan H., Xiang Y. (2019). Genome-wide identification and expression analyses of the bZIP transcription factor genes in moso bamboo (*Phyllostachys edulis*). *International Journal of Molecular Sciences*.

[48] Krishnamurthy P., Hong J. K., Kim J. A., Jeong M. J., Lee Y. H., Lee S. I. (2015). Genome-wide analysis of the expansin gene superfamily reveals Brassica rapa-specific evolutionary dynamics upon whole genome triplication. *Molecular Genetics and Genomics*.

[49] Fujita Y., Fujita M., Satoh R. (2005). AREB1 is a transcription activator of novel ABRE-dependent ABA signaling that enhances drought stress tolerance in Arabidopsis. *Plant Cell*.

[50] Yamaguchi-Shinozaki K., Shinozaki K. (2006). Transcriptional regulatory networks in cellular responses and tolerance to dehydration and cold stresses. *Annual Review of Plant Biology*.

[51] Guo H., Zhang Y., Wang Z. (2019). Genome-wide identification of WRKY transcription factors in the Asteranae. *Plants*.

[52] Iorizzo M., Cavagnaro P. F., Bostan H., Zhao Y., Zhang J., Simon P. W. (2019). A cluster of MYB transcription factors regulates anthocyanin biosynthesis in carrot (*Daucus carota* L.) root and petiole. *Frontiers in Plant Science*.

[53] Cui Q., Yan X., Gao X., Zhang D. M., He H. B., Jia G. X. (2018). Analysis of WRKY transcription factors and characterization of two Botrytis cinerea-responsive LrWRKY genes from Lilium regale. *Plant Physiology and Biochemistry*.

[54] Ali M. A., Azeem F., Nawaz M. A. (2018). Transcription factors WRKY11 and WRKY17 are involved in abiotic stress responses in Arabidopsis. *Journal of Plant Physiology*.

[55] Ji C., Mao X., Hao J. (2018). Analysis of bZIP transcription factor family and their expressions under salt stress in Chlamydomonas reinhardtii. *International Journal of Molecular Sciences*.

[56] Nijhawan A., Jain M., Tyagi A. K., Khurana J. P. (2008). Genomic survey and gene expression analysis of the basic leucine zipper transcription factor family in rice. *Plant Physiology*.

[57] Wang J., Zhou J., Zhang B., Vanitha J., Ramachandran S., Jiang S. Y. (2011). Genome-wide expansion and expression divergence of the basic leucine zipper transcription factors in higher plants with an emphasis on sorghum. *Journal of Integrative Plant Biology*.

[58] Van de Peer Y., Mizrachi E., Marchal K. (2017). The evolutionary significance of polyploidy. *Nature Reviews. Genetics*.

[59] Ren R., Wang H., Guo C. (2018). Widespread whole genome duplications contribute to genome complexity and species diversity in angiosperms. *Molecular Plant*.

[60] Cao L., Lu X., Zhang P., Wang G., Wei L., Wang T. (2019). Systematic analysis of differentially expressed maize ZmbZIP genes between drought and rewatering transcriptome reveals bZIP family members involved in abiotic stress responses. *International Journal of Molecular Sciences*.

[61] Barah P., Jayavelu N. D., Mundy J., Bones A. M. (2013). Genome scale transcriptional response diversity among ten ecotypes of Arabidopsis thaliana during heat stress. *Frontiers in Plant Science*.

[62] Li X., Duan X., Jiang H. (2006). Genome-wide analysis of basic/helix-loop-helix transcription factor family in rice and Arabidopsis. *Plant Physiology*.

[63] Hu W., Wang L., Tie W. (2016). Genome-wide analyses of the bZIP family reveal their involvement in the development, ripening and abiotic stress response in banana. *Scientific Reports*.

[64] Toh S., McCourt P., Tsuchiya Y. (2012). HY5 is involved in strigolactone-dependent seed germination in Arabidopsis. *Plant Signaling & Behavior*.

[65] Chuang C. F., Running M. P., Williams R. W., Meyerowitz E. M. (1999). The PERIANTHIA gene encodes a bZIP protein involved in the determination of floral organ number in Arabidopsis thaliana. *Genes & Development*.

[66] Gibalova A., Renak D., Matczuk K. (2009). AtbZIP34 is required for Arabidopsis pollen wall patterning and the control of several metabolic pathways in developing pollen. *Plant Molecular Biology*.

[67] Thurow C., Schiermeyer A., Krawczyk S., Butterbrodt T., Nickolov K., Gatz C. (2005). Tobacco bZIP transcription factor TGA2.2 and related factor TGA2.1 have distinct roles in plant defense responses and plant development. *The Plant Journal*.

[68] Chen H., Chen W., Zhou J. (2012). Basic leucine zipper transcription factor OsbZIP16 positively regulates drought resistance in rice. *Plant Science*.

[69] Liu Y., Zheng W., Zhang W. (2015). Photoaffinity labeling of transcription factors by DNA-templated crosslinking. *Chemical Science*.

