# Supplementary figures and images for: Genome-Wide Analysis, Characterization, and Expression Profile of the Basic Leucine Zipper Transcription Factor Family in Pineapple
Source: Int J Genomics. 2020 May 11;2020:3165958. doi: 10.1155/2020/3165958 (PMC7238347; doi:10.1155/2020/3165958)

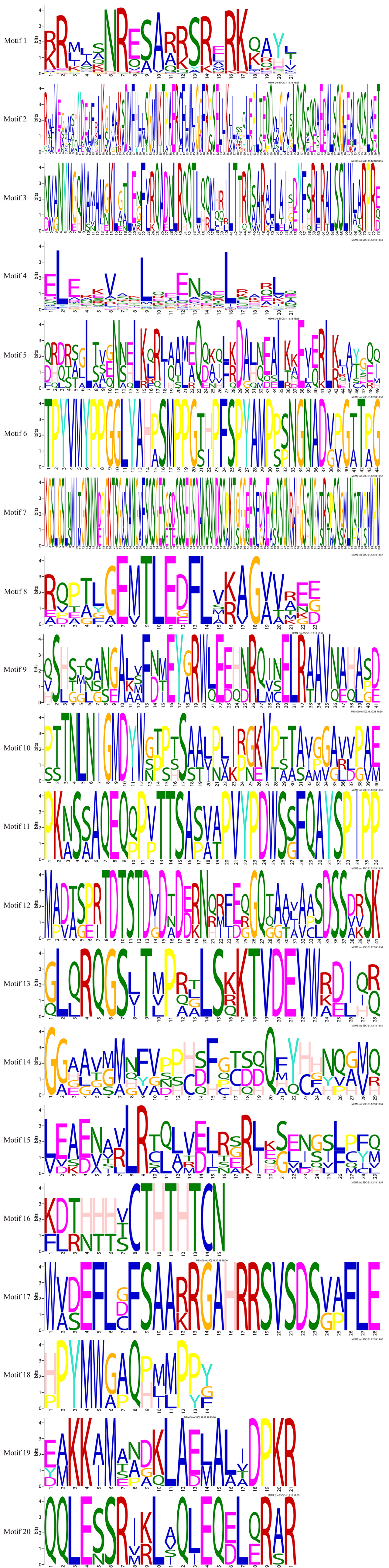

Supplement: Supplementary 1 — Fig. S1: 20 motifs of bZIP proteins in pineapple. [file 3165958.f1.pdf]
